# Gastric Cancer Risk in Patients with Intestinal Metaplasia: Long-Term Outcomes from a Large Single-Center Cohort in Türkiye

**DOI:** 10.3390/jcm14217662

**Published:** 2025-10-28

**Authors:** Veysel Baran Tomar, Ali Karataş, Azar Abiyev, Haluk Cihad Albayrak, Serkan Dumanlı, Serhat Haliloğlu, Efkan Öz, Mehmet Arda İnan, Mehmet Cindoruk, Tarkan Karakan, Murat Kekilli

**Affiliations:** 1 Division of Nephrology, Department of Internal Medicine, Gazi University Faculty of Medicine, 06500 Ankara, Türkiye; 2 Division of Gastroenterology, Department of Internal Medicine, Gazi University Faculty of Medicine, 06500 Ankara, Türkiye; alikaratas@gazi.edu.tr (A.K.); drmehmetcindoruk@gmail.com (M.C.); tkarakan@gmail.com (T.K.); muratkekilli@gazi.edu.tr (M.K.); 3 Department of Gastroenterology, Lokman Hekim University, 06510 Ankara, Türkiye; drazerabiyev@gmail.com; 4 Department of Gastroenterology, Atatürk Sanatoryum Training and Research Hospital, 06280 Ankara, Türkiye; halukcihad23@gmail.com; 5 Department of Gastroenterology, Hatay Training and Research Hospital, 31060 Hatay, Türkiye; s87dumanli@hotmail.com; 6 Department of Internal Medicine, Gazi University Faculty of Medicine, 06500 Ankara, Türkiye; serhathaliloglu@msn.com (S.H.); efkan614@gmail.com (E.Ö.); 7 Department of Pathology, Acıbadem University, 34752 İstanbul, Türkiye; drmardainan@gmail.com

**Keywords:** gastric intestinal metaplasia (GIM), gastric cancer (GC), *Helicobacter pylori*, premalignant gastric lesions, correa cascade, surveillance, risk stratification

## Abstract

**Background/Objectives:** Gastric intestinal metaplasia (GIM) is a recognized premalignant condition for gastric cancer (GC), but long-term outcomes and predictors of progression remain incompletely understood. This study aimed to evaluate the progression of GIM and identify factors associated with malignant transformation. **Methods:** In this retrospective single-center study, 1204 adult patients with histologically confirmed GIM and at least 12 months of follow-up after esophagogastroduodenoscopy (EGD) were analyzed. Clinical and pathological variables, including GIM extent, *Helicobacter pylori* status, family history of GC, demographic factors, and residence in endemic regions, were assessed. Patients were stratified into high- and low-risk groups according to established criteria, and progression to GC or other neoplasms was recorded. **Results:** During a mean follow-up of 38.6 months, 49.1% of patients had no detectable GIM at the end of follow-up, 48.7% remained unchanged, and 2.2% showed disease progression. Among progressed cases, adenocarcinoma accounted for 66.7%, dysplasia for 29.6%, and SCC for 3.7%. Progression was significantly more common among males, older patients, and those with antrum + corpus involvement. The overall progression rate from GIM to adenocarcinoma was 1.5% (approximately 0.45% per patient-year). No significant difference in progression or survival was observed between high- and low-risk groups. **Conclusions:** The long-term malignant transformation rate of GIM is low. Male sex and extensive gastric involvement were associated with higher progression rates, while *H. pylori* was not predictive of malignant transformation. These findings support individualized surveillance strategies for patients with GIM, while routine surveillance of antrum-limited GIM may provide minimal benefit but increase healthcare burden.

## 1. Introduction

Gastric cancer (GC) remains a major global health challenge, ranking as the fourth leading cause of cancer-related mortality worldwide [1]. Its incidence exhibits pronounced geographic variability, with the highest burdens observed in East Asia, Eastern Europe, and parts of Latin America [2]. This heterogeneity reflects complex interactions between genetic susceptibility, environmental exposures, and infectious agents [3].

Most gastric adenocarcinomas evolve through a multistep histopathological sequence that is often clinically silent in its early stages, offering a critical window for surveillance. This process, classically described as the *Correa cascade*, involves the transition from normal gastric mucosa to chronic gastritis, atrophic gastritis (AG), multifocal AG (MAG), gastric intestinal metaplasia (GIM), low-grade or high-grade dysplasia (LGD/HGD), and ultimately invasive adenocarcinoma [3]. Among known risk factors, *H. pylori* infection is predominant, accounting for approximately 75–89% of non-cardia gastric adenocarcinomas [4].

AG, GIM, and dysplasia are recognized as gastric premalignant conditions (GPMCs). GIM is histologically defined as the replacement of oxyntic or antral gastric mucosa by an intestinal-type epithelium containing goblet cells, absorptive cells, and Paneth cells [5]. The likelihood of GIM development and its progression to GC is influenced by multiple determinants, including *H. pylori* exposure history, ethnicity, migration status, age, family history, and environmental cofactors [6]. Reported annual progression rates to GC vary considerably: approximately 0.25% in Western European cohorts compared with up to 10% in certain East Asian populations [7,8].

In high-incidence countries, particularly across Asia, secondary prevention strategies combining endoscopic screening and targeted surveillance of premalignant lesions have been implemented to enable earlier detection and improve prognosis [3]. Nevertheless, the absence of harmonized international guidelines, coupled with the scarcity of large-scale longitudinal studies, limits the global generalizability of these approaches. Existing research on surveillance intervals for GIM has often involved small patient cohorts, and the optimal duration and intensity of follow-up remain a matter of debate.

The present study aims to evaluate the long-term outcomes of GIM and to identify factors that may facilitate its progression to gastric cancer. By considering variables such as GIM extent, presence of additional risk factors, age, and sex, we sought to provide a comprehensive risk assessment for malignant transformation. To our knowledge, this is the largest single-center cohort from Türkiye evaluating GIM progression, thereby offering novel insights from a region with an intermediate incidence of gastric cancer.

## 2. Materials and Methods

### 2.1. Study Design and Setting

This single-center, multidisciplinary, retrospective archival study was conducted at the Division of Gastroenterology, Department of Internal Medicine, and the Department of Pathology, Gazi University Faculty of Medicine, Ankara, Türkiye. The primary objective was to assess the risk of gastric cancer progression among patients with GIM based on lesion distribution, presence of additional risk factors, age, and sex. A secondary objective was to determine the prevalence of *H. pylori*, GIM, gastric adenocarcinoma, gastrointestinal stromal tumors (GIST), and neuroendocrine tumors (NET) among patients undergoing esophagogastroduodenoscopy (EGD) with biopsy.

### 2.2. Study Population and Data Sources

All patients aged ≥18 years who underwent EGD for any indication at Gazi University Hospital between January 2013 and December 2021 were screened. Endoscopic and histopathological records were retrieved from the institutional electronic medical record system and pathology archives.

A total of 25,130 patients underwent EGD during the study period. The Sydney protocol for gastric endoscopic biopsies was routinely followed and reported for all cases in the study. Of these, 3368 patients were histologically diagnosed with GIM based on gastric biopsy specimens. The diagnosis of GIM was confirmed by experienced gastrointestinal pathologists using hematoxylin-eosin staining, with ancillary stains (e.g., Alcian blue, PAS) applied when necessary.

### 2.3. Inclusion and Exclusion Criteria

For the longitudinal risk assessment, only patients with histologically confirmed GIM who had at least one follow-up EGD with biopsy performed ≥12 months after the index procedure were eligible. Patients with incomplete records, prior gastric cancer diagnosis, autoimmune gastritis, or follow-up shorter than one year were excluded. Patients who did not attend scheduled follow-up endoscopies or whose follow-up intervals were inconsistent were also excluded to ensure standardized longitudinal assessment.

### 2.4. Primary and Secondary Endpoints

The primary endpoint of this study was the development of gastric adenocarcinoma during the follow-up period. A secondary, composite endpoint was defined as any histological progression within the Correa cascade (including transition from non-atrophic gastritis to atrophy, intestinal metaplasia, dysplasia, or carcinoma). These definitions were applied consistently across all analyses.

### 2.5. Final Cohort

**Figure 1:**
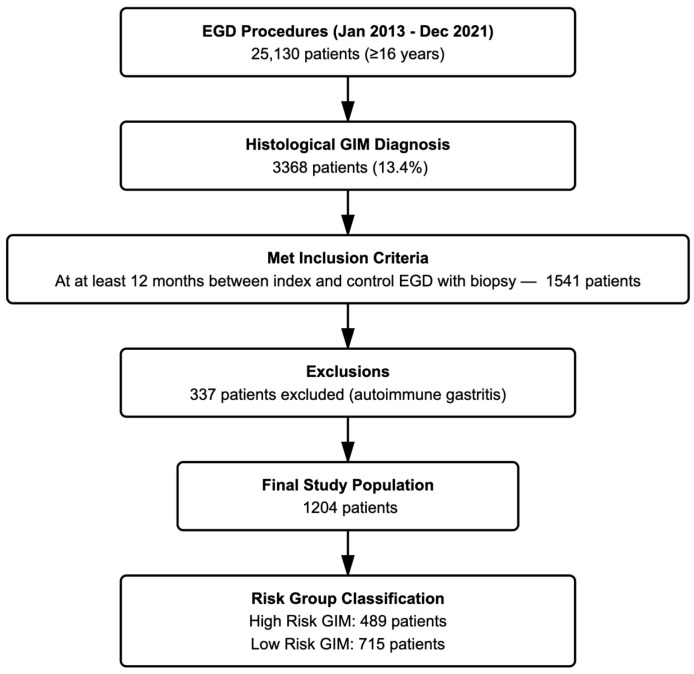
Flowchart of study population.

Out of 3368 patients diagnosed with GIM between 2013 and 2021, 1204 (35.7%) fulfilled the eligibility criteria and constituted the final study cohort (Figure 1). At baseline, the median age was 57 years and 56.9% were female. Limited GIM, confined to the antrum/incisura, was observed in 87.6% of patients, while 12.4% had extensive involvement including the corpus. *H. pylori* infection was detected in 23.9% of cases, and 22.3% reported a family history of gastric cancer. The overall loss to follow-up rate was therefore approximately 64.3%. A detailed breakdown of baseline characteristics is presented in Section 3.

### 2.6. Variables and Definitions

- GIM extent: classified as confined to the antrum, confined to the corpus, or involving both antrum and corpus.
- Additional risk factors: presence of *H. pylori* infection at baseline (determined histologically), family history of gastric cancer in a first-degree relative, residence in a region of Türkiye endemic for gastric cancer (Eastern Anatolian region) [9], demographic characteristics (age, sex), and other documented risk modifiers.
- İntestinal metaplasia risk groups: as outlined in the AJG guidelines, patients with GIM were stratified into two risk groups: the low-risk group, comprising individuals with GIM confined to the antrum; and the high-risk group, which included patients with GIM involving both the antrum and corpus, those with a first-degree relative diagnosed with gastric cancer, and individuals residing in endemic regions [3].
- Outcome: development of histologically confirmed gastric adenocarcinoma during follow-up. Follow-up outcomes of GIM were categorized into three groups: Regression, defined as the histological absence of GIM in follow-up biopsies. Unchanged, defined as the continued presence of GIM without histological progression. Progressed, defined as the development of dysplasia, adenocarcinoma, or other neoplastic lesions during follow-up.

### 2.7. Ethical Approval

The study was conducted in accordance with the principles of the Declaration of Helsinki and was approved by the Gazi University Faculty of Medicine Medical Ethics Committee (Decision No. 343, dated 24 April 2023).

### 2.8. Statistical Analysis

All statistical analyses were performed using SPSS software, version 22.0 (IBM Corp., Armonk, NY, USA). Descriptive statistics were expressed as frequencies and percentages for categorical variables, and as means ± standard deviation or medians (minimum–maximum) for continuous variables, as appropriate. The normality of variable distributions was assessed both visually (histograms and probability plots) and analytically (Kolmogorov–Smirnov/Shapiro–Wilk tests). Comparisons between independent groups were performed using Pearson’s chi-square and Fisher’s exact tests for categorical variables, and one-way ANOVA or Student’s *t*-test for continuous variables. When significant differences were detected among groups, post hoc analyses were conducted using the Bonferroni correction. A two-tailed *p*-value < 0.05 was considered statistically significant.

## 3. Results

### 3.1. Patient Flow and Baseline Characteristics

A total of 25,130 patients aged ≥18 years underwent esophagogastroduodenoscopy (EGD) between January 2013 and December 2021. GIM was histologically diagnosed in 3368 patients (13.4%). Among these, 1541 patients met the inclusion criteria for follow-up analysis, defined as having at least 12 months between the index and control EGD with biopsy. Of these, 337 patients were excluded due to a diagnosis of autoimmune gastritis. Consequently, 1204 patients were included in the study. Among them, 489 patients belonged to the high-risk GIM group, while 715 were classified as low-risk GIM.

The median age of the study cohort was 57 years (range: 18–91), and 56.9% were female. Limited GIM, defined as GIM confined to the antrum and incisura, was present in 87.6% of patients, whereas 12.4% had anatomically extensive GIM involving the corpus. *H. pylori* infection was detected in 23.9% of patients at baseline. Additional risk factors included a family history of gastric cancer in a first-degree relative (22.3%) and residence in a region of Türkiye endemic for gastric cancer (11.6%).

### 3.2. Follow-Up Outcomes of GIM Patients

The mean follow-up period of patients followed with GIM was 38.6 ± 26.7 months. Among 1204 patients, 49.1% (n = 591) had no detectable GIM at the end of follow-up, 48.7% (n = 586) showed no change, and 2.2% (n = 27) demonstrated disease progression.

**Figure 2:**
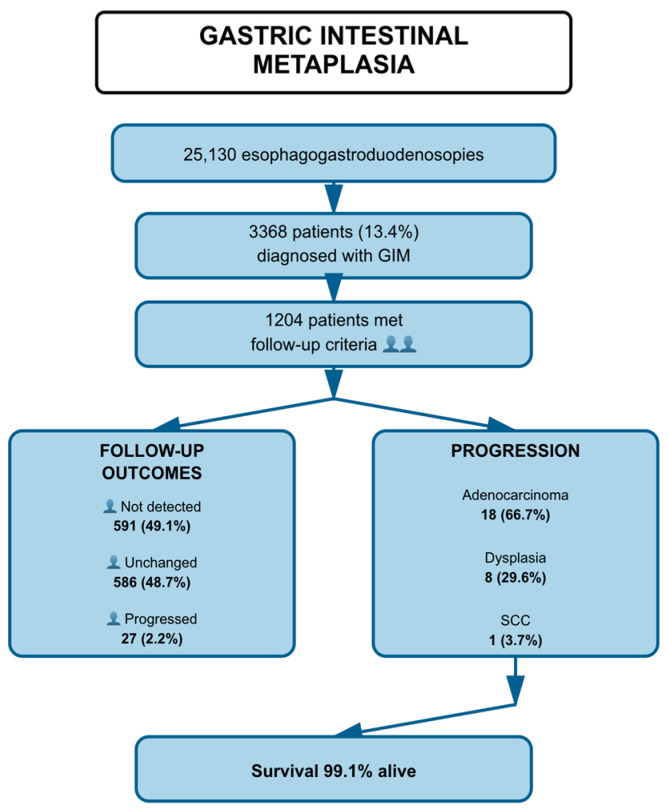
Follow-up outcomes and progression patterns in patients with GIM.

Among the 27 patients with documented progression, the most common pathological outcome was adenocarcinoma (66.7%, n = 18), followed by dysplasia (29.6%, n = 8), and SCC (3.7%, n = 1) (Figure 2).

The progression rate from GIM to adenocarcinoma was 18 out of 1204 patients (1.5%) over a mean follow-up of 38.6 months, corresponding to an annual incidence of approximately 0.45% per patient-year.

Regarding survival status, 99.1% (n = 1193) of patients were alive at the last follow-up, while 0.9% (n = 11) had died (Table 1).

Nearly half of the patients showed regression of GIM, while only 2.9% progressed during follow-up.

Table 2 summarizes the distribution of follow-up outcomes according to clinical and pathological factors in 1204 patients with GIM. Statistically significant associations were observed between follow-up status and gender, location of involvement and age (all *p* < 0.05). Disease progression was significantly more frequent among males, older age and antrum + corpus location.

Progression was significantly more common among males, older patients, and those with antrum + corpus involvement.

A significant association was found between follow-up outcome and survival in patients with GIM (*p* = 0.001). Mortality was significantly higher in patients who showed disease progression during follow-up (9.8%) compared to those with unchanged or no detectable GIM (0.4% and 0.9%, respectively) (Table 3).

Among 262 patients diagnosed with adenocarcinoma, 83.2% had adenocarcinoma detected at the initial EGD. Prior GIM diagnosis was noted in 8.1% of these patients before progression to adenocarcinoma, while 8.7% had no premalignant or malignant lesions identified in previous endoscopies.

Mortality was substantially higher in the progression group compared to patients without progression.

### 3.3. Disease Progression and Survival Outcomes by Risk Group

Among 1204 patients categorized into high- and low-risk groups, significant association was found between risk level and disease progression (*p* = 0.004). Progression rates were 2.7% in the low-risk group and 1.6% in the high-risk group (Table 4).

Progression rates were significantly different between high- and low-risk groups.

Among the 36 patients who exhibited disease progression during follow-up, the distribution of pathological diagnoses was assessed according to risk group. In both the high-risk (n = 12) and low-risk (n = 24) groups, adenocarcinoma was the most common diagnosis (31.6% vs. 68.4%), followed by neuroendocrine tumors (NET) and dysplasia. Less frequent diagnoses included gastrointestinal stromal tumor (GIST) and lymphoma, both occurring exclusively in the low-risk group.

Statistical analysis revealed no significant difference in the distribution of pathological diagnoses between high- and low-risk groups (*p* = 0.901), indicating that risk classification was not associated with a specific pathological outcome among patients who progressed (Table 5).

Adenocarcinoma was the predominant progression outcome in both risk groups.

**Figure 3:**
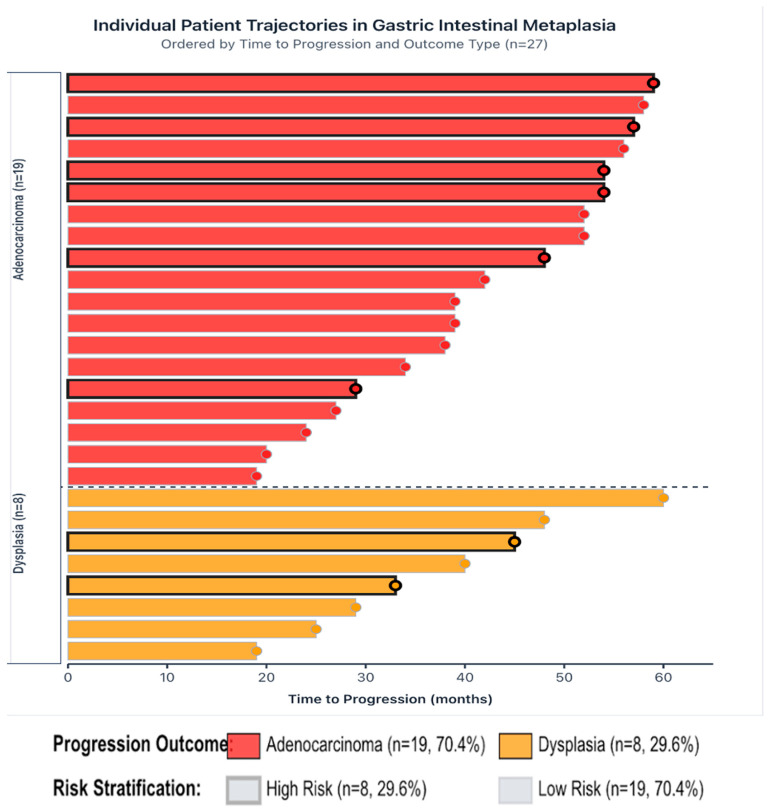
Individual patient trajectories in GIM. Each horizontal bar represents one patient, with bar length corresponding to the time (in months) from the initial diagnosis to the first documented histological progression. Color coding indicates progression type: red bars represent progression to adenocarcinoma (n = 19, 70.4%), and orange bars represent progression to dysplasia (n = 8, 29.6%). Border thickness denotes risk stratification: thick black borders indicate high-risk patients (n = 8, 29.6%), and thin gray borders indicate low-risk patients (n = 19, 70.4%).

Swimmer plot illustrating individual patient trajectories from baseline endoscopy to disease progression among 27 patients with GIM (Figure 3).

Survival analysis showed no statistically significant difference between high- and low-risk groups (*p* = 0.386). Mortality rates were low in both groups, with 0.6% in the high-risk and 1.1% in the low-risk groups (Table 6).

**Figure 4:**
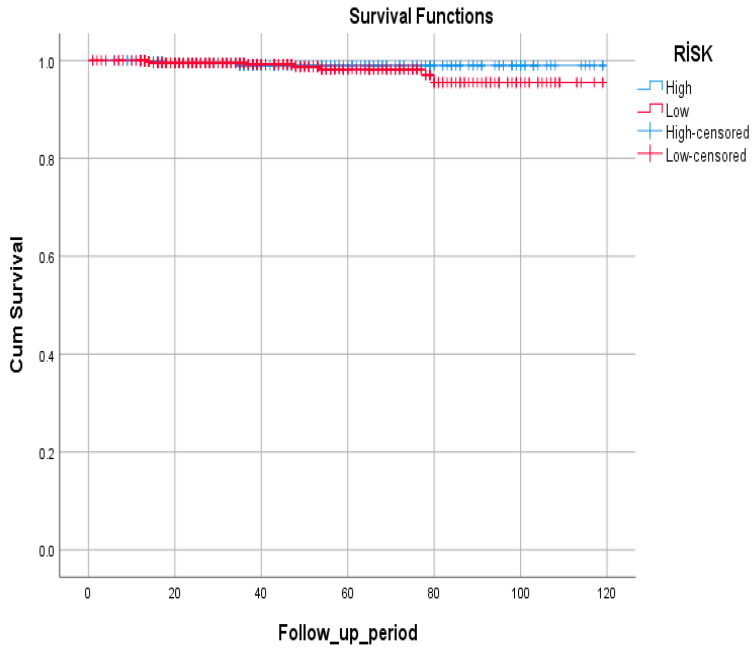
Kaplan–Meier survival curves of patients with GIM according to risk stratification (high-risk vs. low-risk groups). Time axis represents follow-up duration in months. No significant difference in overall survival was observed between groups (log-rank *p* = 0.386).

Survival rates were similarly high in both high- and low-risk groups (Figure 4).

## 4. Discussion

The prevalence of GIM in a given population is largely influenced by the rate of *H. pylori* infection. A Northern European study reported an GIM prevalence of 19% [10]. In our cohort of 25,130 individuals undergoing EGD, GIM was detected in 13.4%, a figure consistent with a study from Van, Turkey (2010–2014), which reported a prevalence of 13.8% among 4050 patients. Determining the true prevalence remains challenging due to the asymptomatic nature of GIM [11].

In large-scale cohort studies, such as those from the Netherlands (1991–2004), the annual progression rate of GIM to adenocarcinoma ranged from 0.1% to 0.9% [12]. In our cohort, the progression rate from GIM to adenocarcinoma was 1.5% over a mean follow-up of 38.6 months (annual incidence ~0.45% per patient-year). This rate is consistent with low-risk Western cohorts but remains lower than those reported from high-incidence Asian populations [13].

Gender differences play a role in gastric cancer pathogenesis. Data from the United States indicate that men are more likely to develop proximal gastric cancer [14], and global age-standardized rates are approximately twice as high in men as in women (12.8% vs. 5.7%) [15]. In the United Kingdom, the male-to-female ratio declines after age 50–55, likely due to reduced protective hormonal effects in postmenopausal women [16]. Long-term estrogen exposure has been associated with a lower gastric cancer risk [17]. In line with previous studies, we observed higher malignant progression rates in men with GIM (3.3%) compared to women (2.6%).

*H. pylori* infection, a key initiator in the Correa cascade, affects an estimated 4.4 billion people worldwide [18]. In the United Kingdom, 32% of gastric cancers are attributable to *H. pylori* [19]. The World Health Organization (WHO) classifies *H. pylori* as a class I carcinogen [20,21]. However, in advanced gastritis and GIM, mucosal changes may render the gastric environment inhospitable to *H. pylori*, leading to spontaneous bacterial clearance [22]. In our study, *H. pylori* was detected in 23.9% of GIM patients, and progression was more common in *H. pylori*-negative individuals (3.2%) compared to *H. pylori*-positive individuals (1.9%). Whether this reflects prior eradication therapy could not be determined.

Histologically, GIM can be classified into three subtypes (Types 1–3) according to mucin staining [23,24,25], with incomplete Type 3 GIM showing the strongest association with gastric cancer [26,27]. Due to technical challenges and the use of toxic reagents, such staining is now largely restricted to research settings [28], and our study did not perform histologic subtyping.

ECL cell hyperplasia, often associated with autoimmune gastritis, was present in 5.6% of our GIM patients. Progression occurred in 6.1% of these cases compared to 3.4% without ECL hyperplasia, though no adenocarcinoma developed in the hyperplasia group. The lack of statistical association between ECL hyperplasia and adenocarcinoma aligns with its stronger link to type I neuroendocrine tumors [29,30,31,32,33,34].

Anatomically, GIM is categorized as limited (antrum or incisura only) or extensive (involving corpus and additional sites) [35,36,37,38]. Prior studies suggest that extent of involvement may be more prognostically relevant than histological subtype [39]. In our series, progression rates were highest for extensive involvement (5.5%), followed by limited antrum involvement only (3.2%). Adenocarcinoma was significantly more common in cases involving the corpus or both corpus and antrum (*p* < 0.05). While our findings suggest that routine surveillance may not be necessary for antrum-limited GIM, this conclusion should be interpreted with caution given the low number of progression events and limited statistical power.

Globally, there is no uniform consensus on GIM surveillance. High-incidence countries such as Japan and South Korea recommend population-based endoscopic screening, with national programs in place since the early 2000s [40,41]. In low-incidence European countries, the European Society of Gastrointestinal Endoscopy (ESGE) advises three-year follow-up for patients with GIM in both antrum and corpus, but no surveillance for antrum-only disease [42].

According to the latest ACG guidelines, patients with GIM are categorized into two groups for surveillance: high-risk and low-risk [3]. The high-risk group includes patients with extensive involvement, those with a first-degree relative with gastric cancer, and individuals from regions endemic for gastric cancer. In our study, patients were similarly stratified. Patients born in Eastern Anatolia, a gastric cancer endemic region in our country, were included in the high-risk group [9]. Interestingly, in our cohort the low-risk group demonstrated a slightly higher progression rate compared to the high-risk group (2.7% vs. 1.6%, *p* = 0.004), although survival outcomes did not differ. This paradoxical finding may partly reflect the absence of histological subtyping (complete vs. incomplete GIM), since incomplete GIM is known to carry a higher malignant potential and its distribution between groups was not captured. In addition, the use of geographic endemicity as a risk criterion may have led to misclassification in a population with frequent historical internal migration, thereby obscuring true risk patterns.

Several limitations should be acknowledged. First, this study was retrospective, which may introduce selection and information biases. In particular, heterogeneous follow-up intervals and variability in biopsy sampling protocols could have influenced the observed outcomes. Second, histological subtyping of GIM (Types 1–3) was not performed, limiting the assessment of subtype-specific risk. Incomplete subtyping of GIM was not performed, which is an important limitation since incomplete GIM is associated with a higher risk of malignant transformation. Third, *H. pylori* eradication history and post-treatment confirmation were not systematically available. Therefore, interpretation of *H. pylori* negativity should be made with caution, as some cases may represent prior successful eradication or spontaneous clearance in advanced atrophic gastritis. This limitation introduces the potential for misclassification bias and restricts firm conclusions regarding the role of *H. pylori* status in progression. Fourth, although follow-up data were comprehensive, the relatively low number of progression events limits the statistical power for certain subgroup analyses. Additionally, the relatively high regression rate observed in our cohort should be interpreted with caution, as it may reflect both true histological reversal and potential biopsy sampling variability. This possibility has been reported in prior studies and represents an inherent limitation of observational GIM cohorts. Fifth, patients with autoimmune gastritis were excluded, which might have led to underestimation of the true incidence of neuroendocrine tumors in this population. Sixth, loss to follow-up and exclusion of patients without adequate surveillance endoscopies may have introduced a selection bias, potentially underestimating progression risk. The small number of events and incomplete survival curves precluded estimation of median survival or RMST. Therefore, mean survival times were reported, which more appropriately represent the available follow-up information in this cohort. Finally, as a single-country study, our findings may not be generalizable to populations with different *H. pylori* prevalence, genetic backgrounds, or gastric cancer incidence.

## 5. Conclusions

In our large cohort study, GIM was detected in 13.4% of patients undergoing esophagogastroduodenoscopy, with an overall cumulative progression rate to adenocarcinoma of 1.5% during a mean follow-up of 38.6 months (annual incidence ~0.45% per patient-year). Male sex and extensive gastric involvement were associated with higher progression rates. *H. pylori* infection, while important in the pathogenesis of gastric carcinogenesis, was not predictive of malignant transformation in our cohort, possibly due to prior eradication therapy or spontaneous clearance in advanced lesions. Our findings also suggest that heterogeneous risk profiles, including birth in gastric cancer endemic regions, family history, and extent of GIM, may obscure clear stratification between high- and low-risk groups. These results support the need for individualized surveillance strategies based on anatomical extent of GIM and patient-specific risk factors rather than *H. pylori* status alone. Routine surveillance of patients with GIM limited to the antrum appears to provide minimal clinical benefit while imposing unnecessary procedural burden and healthcare costs.

## Figures and Tables

**Table 1:** Outcomes and Survival Status of Patients Followed with GIM.

Category	Variable	n	%
Follow-up Outcome (n = 1204)	Not detected	591	49.1
	Unchanged	586	48.7
	Progressed	27	2.2
Pathology Results in Progressed Cases (n = 27)	Adenocarcinoma	18	66.7
	Dysplasia	8	29.6
	SCC	1	3.7
Survival Status (n = 1204)	Alive	1193	99.1
	Deceased	11	0.9

Note: Percentages are reported as column percentages.

**Table 2:** Factors Associated with Follow-up Outcomes in Patients with GIM (n = 1204).

Variable	Category	Not Detected n (%)/Mean ± SD	Unchanged n (%)/Mean ± SD	Progressed n (%)/Mean ± SD	*p*
Gender	Female	372 (54.0)	299 (43.4)	18 (2.6)	**<0.001**
	Male	219 (42.5)	279 (54.2)	17 (3.3)	
Location of Involvement	Antrum	540 (52.8)	455 (44.5)	28 (2.7)	**<0.001**
	Antrum + Corpus	51 (28.2)	123 (68.0)	7 (3.9)	
ECL Hyperplasia	Present	12 (44.4)	15 (55.6)	0 (0.0)	0.747
	Absent	579 (49.2)	563 (47.8)	35 (3.0)	
*H. pylori* Presence	Present	162 (52.6)	110 (45.5)	6 (1.9)	0.233
	Absent	429 (47.9)	438 (48.9)	29 (3.2)	
Age (years)	—	54.8 ± 12.4	58.3 ± 11.0	60.5 ± 11.6	**<0.001**
Follow-up Duration (months)	—	39.3 ± 26.2	38.3 ± 27.2	31.2 ± 24.9	0.111

**Table 3:** Survival Outcomes by Follow-up Status and Initial EGD Findings in Patients with GIM.

Variable	Category	Alive n (%)	Deceased n (%)	*p*-Value
Survival by Follow-up Status (n = 1204)	Not Detected	587 (99.3)	4 (0.7)	**<0.001**
	Unchanged	583 (99.5)	3 (0.5)	
	Progressed	23 (85.2)	4 (14.8)	

**Table 4:** Progression outcomes of GIM risk groups.

Risk Group	Not Detected	Unchanged	Progressed	Total	*p* Value
High	215 (44.0%)	266 (54.4%)	8 (1.6%)	489 (40.6%)	**0.004**
Low	376 (52.6%)	320 (44.8%)	19 (2.7%)	715 (59.4%)	
Total	591 (49.1%)	586 (48.7%)	27 (2.2%)	1204 (100.0%)	

**Table 5:** Summary of adverse outcomes during follow-up.

Pathology Diagnosis	Total n (%)	High Risk n (%)	Low Risk n (%)
Adenocarcinoma	19 (52.8%)	6 (31.6%)	13 (68.4%)
NET (Neuroendocrine Tumor)	5 (13.9%)	4 (80.0%)	1 (20.0%)
Dysplasia	8 (22.2%)	2 (25.0%)	6 (75.0%)
GIST (Gastrointestinal Stromal Tumor)	3 (8.3%)	0 (0.0%)	3 (100.0%)
Lymphoma	1 (2.8%)	0 (0%)	1 (100.0%)
Total	36 (100%)	12 (100%)	24 (100%)

**Table 6:** Survival outcomes of GIM risk groups.

Risk Group	Alive	Deceased	Total
High	486 (99.4%)	3 (0.6%)	489
Low	707 (98.9%)	8 (1.1%)	715
Total	1193 (99.1%)	11 (0.9%)	1204

## Data Availability

The data that support the findings of this study are available from the corresponding author upon reasonable request.
